# *catena*-Poly[hemi(hexane-1,6-diammonium) [[aqua­dibromido­manganese(II)]-μ-pyridine-2-carboxyl­ato]]

**DOI:** 10.1107/S1600536809016316

**Published:** 2009-05-07

**Authors:** Nam-Ho Kim, In-Chul Hwang, Kwang Ha

**Affiliations:** a School of Applied Chemical Engineering, The Research Institute of Catalysis, Chonnam National University, Gwangju 500-757, Republic of Korea; b Institute of Basic Sciences, Pohang University of Science and Technology, Pohang 790-784, Republic of Korea

## Abstract

The asymmetric unit of the title compound, {(C_6_H_18_N_2_)_0.5_[MnBr_2_(C_6_H_4_NO_2_)(H_2_O)]}_*n*_, contains the repeat unit of the complex anion and one-half of a hexane-1,6-diammonium cation that is located on a twofold rotation axis. In the anionic polymer, the Mn^2+^ ions are bridged by the pyridine­carboxyl­ate (pic) anion ligand, forming a chain structure along the *c* axis. The Mn^2+^ ion is six-coordinated in a distorted octa­hedral environment by one N atom of the pyridine ring, two O atoms of the two carboxyl­ate groups, one O atom of the water mol­ecule and two Br atoms. The compound displays inter­molecular N—H⋯O, N—H⋯Br, O—H⋯Br and O—H⋯O hydrogen bonding. There may also be inter­molecular π–π inter­actions between adjacent pyridine rings, with a centroid–centroid distance of 3.992 (4) Å.

## Related literature

For the synthesis and structure of the Mn(III)–pic complex, [Mn(pic)_3_], see: Figgis *et al.* (1978 ▶); Yamaguchi & Sawyer (1985 ▶); Li *et al.* (2000 ▶). For the synthesis and structure of the Mn(II)–pic complex, [Mn(pic)_2_(H_2_O)_2_], see: Okabe & Koizumi (1998 ▶); Barandika *et al.* (1999 ▶). For details of mono-, di- and polynuclear Mn(II, III, IV)–pic complexes, see: Huang *et al.* (2004 ▶).
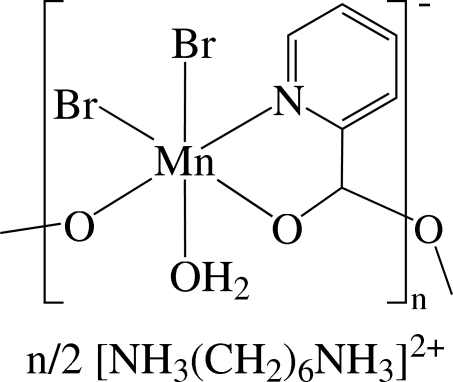


## Experimental

### 

#### Crystal data

(C_6_H_18_N_2_)_0.5_[MnBr_2_(C_6_H_4_NO_2_)(H_2_O)]
*M* *_r_* = 413.99
Monoclinic, 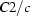

*a* = 13.490 (3) Å
*b* = 21.510 (5) Å
*c* = 9.803 (2) Å
β = 91.125 (4)°
*V* = 2843.9 (11) Å^3^
*Z* = 8
Mo *K*α radiation
μ = 6.55 mm^−1^
*T* = 293 K
0.10 × 0.10 × 0.10 mm

#### Data collection

Bruker SMART 1000 CCD diffractometer
Absorption correction: multi-scan (*SADABS*; Bruker, 2000 ▶) *T*_min_ = 0.394, *T*_max_ = 0.520
7815 measured reflections
2705 independent reflections
1846 reflections with *I* > 2σ(*I*)
*R*_int_ = 0.030

#### Refinement

*R*[*F* ^2^ > 2σ(*F* ^2^)] = 0.031
*wR*(*F* ^2^) = 0.114
*S* = 0.94
2705 reflections
152 parameters
H-atom parameters constrained
Δρ_max_ = 0.68 e Å^−3^
Δρ_min_ = −0.56 e Å^−3^

### 

Data collection: *SMART* (Bruker, 2000 ▶); cell refinement: *SAINT* (Bruker, 2000 ▶); data reduction: *SAINT*; program(s) used to solve structure: *SHELXS97* (Sheldrick, 2008 ▶); program(s) used to refine structure: *SHELXL97* (Sheldrick, 2008 ▶); molecular graphics: *PLATON* (Spek, 2009 ▶); software used to prepare material for publication: *SHELXL97*.

## Supplementary Material

Crystal structure: contains datablocks global, I. DOI: 10.1107/S1600536809016316/cs2117sup1.cif

Structure factors: contains datablocks I. DOI: 10.1107/S1600536809016316/cs2117Isup2.hkl

Additional supplementary materials:  crystallographic information; 3D view; checkCIF report

## Figures and Tables

**Table 1:** Hydrogen-bond geometry (Å, °)

*D*—H⋯*A*	*D*—H	H⋯*A*	*D*⋯*A*	*D*—H⋯*A*
N2—H2*A*⋯O1^i^	0.89	2.44	2.949 (6)	117
N2—H2*A*⋯O2^i^	0.89	2.47	3.300 (6)	155
N2—H2*B*⋯Br2^i^	0.89	2.61	3.339 (4)	139
N2—H2*C*⋯Br2^ii^	0.89	2.58	3.417 (5)	157
O3—H3*A*⋯Br1^iii^	0.99	2.28	3.245 (4)	165
O3—H3*B*⋯O1^iv^	0.83	2.18	2.961 (5)	157

Symmetry codes: (i) 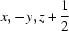; (ii) 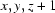; (iii) 
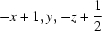
; (iv) 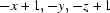.

**Fig. 1.**
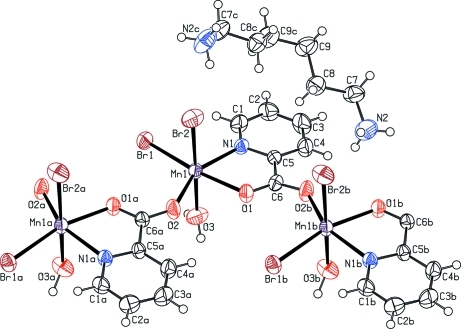
A structure detail of the title compound, with displacement ellipsoids drawn at the 50% probability level for non-H atoms [Symmetry codes: (a) x, -y, -1/2 + z, (b) x, -y, 1/2 + z, (c) -x, y, 3/2 - z].

**Fig. 2.**
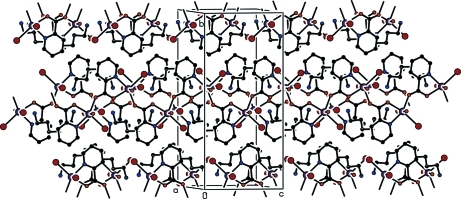
View of the unit-cell contents and chain structure of the title compound. H atoms have been omitted for clarity.

## supplementary crystallographic information

### Comment

Complex polymers are attracting great attention because of their potential applications such as in catalysis, magnetism, molecular recognition and other fileds (Huang *et al.*, 2004). The title compound, {(C_6_H_18_N_2_)_0.5_[MnBr_2_(C_6_H_4_NO_2_)(H_2_O)]}_n_, consists of an anionic complex chain polymer with counter-cations (Fig. 1). In the anionic polymer, symmetry related Mn^2+^ ions are bridged by pyridinecarboxylate (pic) anion ligands to form one-dimensional zigzag chain structures along the *c* axis (Fig. 2). The Mn ion is six-coordinated in a distorted octahedral structure by one N atom of the pyridine ring, two O atoms of two carboxylate groups, one O atom of the water molecule and two Br atoms. The three O atoms are disposed in the facial position. The asymmetric unit contains the repeat unit of the polymer, [MnBr_2_(C_6_H_4_NO_2_)(H_2_O)]^-^, and one half of a 1,6-diammoniohexane cation. Cations sit on a 2-fold symmetry axes at 0, *y*, 1/4 (Wyckoff letter *e*). The compound displays intermolecular hydrogen bonding (Table 1). There may be also intermolecular π-π interactions between adjacent pyridine rings, with a centroid-centroid distance of 3.992 (4) Å. The structure of the anionic complex polymer is very similar to the structure of the neutral compound [MnCl(pic)(H_2_O)_2_]_n_ in which the Mn ions are linked to each other by pyridinecarboxylate bridges in a *syn - anti* mode (Huang *et al.*, 2004).

### Experimental

A solution of MnBr_2_.4H_2_O (0.116 g, 0.404 mmol), pyridine-2-carboxylic acid (0.101 g, 0.734 mmol) and 1,6-diaminohexane (0.021 g, 0.184 mmol) in H_2_O (10 ml) was refluxed for 4 h. The solvent was removed under vacuum and the residue was dried at 70 °C, to give a pale yellow film. Crystals suitable for X-ray analysis were obtained by slow evaporation from a CH_3_CN solution.

### Refinement

H atoms were positioned geometrically and allowed to ride on their respective parent atoms [C—H = 0.93 Å (aromatic) or 0.97 Å (CH_2_) and N—H = 0.89 Å, and *U*_iso_(H) = 1.2*U*_eq_(C) or 1.5*U*_eq_(N)]. The H atoms of the water molecule were located from Fourier difference maps, but not refined.

### Crystal data

**Table:**

(C_6_H_18_N_2_)_0.5_[MnBr_2_(C_6_H_4_NO_2_)(H_2_O)]	*F*(000) = 1616
*M**_r_* = 413.99	*D*_x_ = 1.934 Mg m^−^^3^
Monoclinic, *C*2/*c*	Mo *K*α radiation, λ = 0.71073 Å
Hall symbol: -C 2yc	Cell parameters from 806 reflections
*a* = 13.490 (3) Å	θ = 2.8–25.3°
*b* = 21.510 (5) Å	µ = 6.55 mm^−^^1^
*c* = 9.803 (2) Å	*T* = 293 K
β = 91.125 (4)°	Stick, colorless
*V* = 2843.9 (11) Å^3^	0.10 × 0.10 × 0.10 mm
*Z* = 8	

### Data collection

**Table:**

Bruker SMART 1000 CCD diffractometer	2705 independent reflections
Radiation source: fine-focus sealed tube	1846 reflections with *I* > 2σ(*I*)
graphite	*R*_int_ = 0.030
φ and ω scans	θ_max_ = 25.7°, θ_min_ = 1.8°
Absorption correction: multi-scan (*SADABS*; Bruker, 2000)	*h* = −16→15
*T*_min_ = 0.394, *T*_max_ = 0.520	*k* = −26→26
7815 measured reflections	*l* = −7→11

### Refinement

**Table:**

Refinement on *F*^2^	Primary atom site location: structure-invariant direct methods
Least-squares matrix: full	Secondary atom site location: difference Fourier map
*R*[*F*^2^ > 2σ(*F*^2^)] = 0.031	Hydrogen site location: inferred from neighbouring sites
*wR*(*F*^2^) = 0.114	H-atom parameters constrained
*S* = 0.94	*w* = 1/[σ^2^(*F*_o_^2^) + (0.0606*P*)^2^] where *P* = (*F*_o_^2^ + 2*F*_c_^2^)/3
2705 reflections	(Δ/σ)_max_ < 0.001
152 parameters	Δρ_max_ = 0.68 e Å^−^^3^
0 restraints	Δρ_min_ = −0.56 e Å^−^^3^

### Fractional atomic coordinates and isotropic or equivalent isotropic displacement parameters (Å^2^)

**Table:**

	*x*	*y*	*z*	*U*_iso_*/*U*_eq_	
Mn1	0.32935 (6)	0.06982 (4)	0.41356 (8)	0.0340 (2)	
Br1	0.34335 (4)	0.15379 (3)	0.21825 (6)	0.0438 (2)	
Br2	0.13026 (5)	0.06928 (3)	0.40001 (8)	0.0585 (2)	
O1	0.3336 (3)	0.01771 (15)	0.6076 (4)	0.0406 (10)	
O2	0.3331 (3)	−0.02393 (17)	0.3343 (4)	0.0491 (11)	
O3	0.4928 (3)	0.06506 (18)	0.4217 (5)	0.0549 (12)	
H3A	0.5358	0.0983	0.3865	0.082*	
H3B	0.5394	0.0408	0.4376	0.082*	
N1	0.3431 (3)	0.14114 (19)	0.5834 (4)	0.0349 (11)	
C1	0.3514 (4)	0.2031 (2)	0.5714 (6)	0.0442 (15)	
H1	0.3488	0.2203	0.4844	0.053*	
C2	0.3634 (5)	0.2422 (3)	0.6809 (7)	0.0519 (16)	
H2	0.3667	0.2850	0.6687	0.062*	
C3	0.3702 (5)	0.2168 (3)	0.8077 (7)	0.0558 (18)	
H3	0.3800	0.2422	0.8836	0.067*	
C4	0.3625 (5)	0.1532 (3)	0.8234 (6)	0.0470 (15)	
H4	0.3667	0.1351	0.9095	0.056*	
C5	0.3488 (4)	0.1175 (2)	0.7091 (5)	0.0318 (12)	
C6	0.3378 (4)	0.0470 (3)	0.7180 (6)	0.0343 (13)	
N2	0.1654 (3)	0.06720 (19)	1.0558 (5)	0.0717 (19)	
H2A	0.2232	0.0622	1.0151	0.107*	
H2B	0.1262	0.0350	1.0366	0.107*	
H2C	0.1754	0.0696	1.1457	0.107*	
C7	0.1177 (3)	0.12511 (19)	1.0062 (5)	0.0564 (18)	
H7A	0.1654	0.1588	1.0122	0.068*	
H7B	0.0630	0.1353	1.0650	0.068*	
C8	0.0796 (5)	0.1202 (3)	0.8624 (7)	0.0545 (17)	
H8A	0.0399	0.0828	0.8525	0.065*	
H8B	0.1351	0.1169	0.8013	0.065*	
C9	0.0174 (5)	0.1762 (3)	0.8230 (7)	0.065 (2)	
H9A	−0.0400	0.1777	0.8810	0.078*	
H9B	0.0560	0.2136	0.8400	0.078*	

### Atomic displacement parameters (Å^2^)

**Table:**

	*U*^11^	*U*^22^	*U*^33^	*U*^12^	*U*^13^	*U*^23^
Mn1	0.0451 (5)	0.0302 (4)	0.0267 (5)	0.0019 (4)	0.0001 (4)	0.0004 (4)
Br1	0.0526 (4)	0.0416 (3)	0.0371 (4)	0.0026 (3)	0.0016 (3)	0.0072 (3)
Br2	0.0466 (4)	0.0593 (4)	0.0697 (5)	0.0030 (3)	0.0012 (3)	0.0179 (4)
O1	0.068 (3)	0.0250 (19)	0.029 (2)	−0.0015 (18)	0.001 (2)	0.0003 (17)
O2	0.082 (3)	0.036 (2)	0.029 (2)	0.003 (2)	−0.001 (2)	−0.0072 (18)
O3	0.042 (2)	0.055 (3)	0.068 (3)	0.008 (2)	0.005 (2)	0.021 (2)
N1	0.045 (3)	0.029 (3)	0.031 (3)	0.002 (2)	0.000 (2)	0.004 (2)
C1	0.062 (4)	0.028 (3)	0.043 (4)	−0.002 (3)	0.001 (3)	0.009 (3)
C2	0.066 (4)	0.029 (3)	0.061 (4)	0.001 (3)	0.003 (4)	−0.005 (3)
C3	0.082 (5)	0.034 (3)	0.051 (4)	−0.004 (3)	0.001 (4)	−0.015 (3)
C4	0.069 (4)	0.037 (3)	0.035 (3)	0.001 (3)	0.000 (3)	−0.001 (3)
C5	0.038 (3)	0.029 (3)	0.028 (3)	0.004 (2)	0.004 (2)	0.001 (2)
C6	0.036 (3)	0.038 (3)	0.030 (3)	−0.002 (2)	−0.001 (3)	0.005 (3)
N2	0.092 (5)	0.050 (3)	0.072 (4)	0.012 (3)	−0.031 (4)	−0.015 (3)
C7	0.051 (4)	0.050 (4)	0.067 (5)	0.009 (3)	−0.016 (4)	−0.012 (4)
C8	0.050 (4)	0.060 (4)	0.053 (4)	0.001 (3)	−0.005 (3)	−0.003 (3)
C9	0.079 (5)	0.042 (4)	0.073 (5)	0.007 (4)	−0.019 (4)	−0.003 (4)

### Geometric parameters (Å, °)

**Table:**

Mn1—O2	2.162 (4)	C4—C5	1.368 (7)
Mn1—O3	2.206 (4)	C4—H4	0.93
Mn1—O1	2.208 (4)	C5—C6	1.526 (7)
Mn1—N1	2.269 (4)	C6—O2^ii^	1.247 (6)
Mn1—Br1	2.6416 (11)	N2—C7	1.4799
Mn1—Br2	2.6864 (12)	N2—H2A	0.89
O1—C6	1.253 (6)	N2—H2B	0.89
O2—C6^i^	1.247 (6)	N2—H2C	0.89
O3—H3A	0.99	C7—C8	1.495 (7)
O3—H3B	0.83	C7—H7A	0.97
N1—C5	1.334 (6)	C7—H7B	0.97
N1—C1	1.343 (6)	C8—C9	1.514 (8)
C1—C2	1.371 (8)	C8—H8A	0.97
C1—H1	0.93	C8—H8B	0.97
C2—C3	1.359 (9)	C9—C9^iii^	1.497 (13)
C2—H2	0.93	C9—H9A	0.97
C3—C4	1.382 (8)	C9—H9B	0.97
C3—H3	0.93		
			
O2—Mn1—O3	86.53 (16)	C5—C4—H4	120.9
O2—Mn1—O1	80.53 (14)	C3—C4—H4	120.9
O3—Mn1—O1	86.35 (15)	N1—C5—C4	123.2 (5)
O2—Mn1—N1	153.14 (15)	N1—C5—C6	115.3 (5)
O3—Mn1—N1	86.42 (16)	C4—C5—C6	121.5 (5)
O1—Mn1—N1	73.17 (14)	O2^ii^—C6—O1	126.0 (5)
O2—Mn1—Br1	112.02 (11)	O2^ii^—C6—C5	117.1 (5)
O3—Mn1—Br1	88.42 (11)	O1—C6—C5	117.0 (5)
O1—Mn1—Br1	166.09 (10)	C7—N2—H2A	109.5
N1—Mn1—Br1	93.65 (11)	C7—N2—H2B	109.5
O2—Mn1—Br2	90.48 (12)	H2A—N2—H2B	109.5
O3—Mn1—Br2	176.99 (12)	C7—N2—H2C	109.5
O1—Mn1—Br2	92.83 (11)	H2A—N2—H2C	109.5
N1—Mn1—Br2	96.13 (12)	H2B—N2—H2C	109.5
Br1—Mn1—Br2	93.02 (3)	N2—C7—C8	112.9 (3)
C6—O1—Mn1	119.3 (3)	N2—C7—H7A	109.0
C6^i^—O2—Mn1	134.6 (4)	C8—C7—H7A	109.0
Mn1—O3—H3A	123.2	N2—C7—H7B	109.0
Mn1—O3—H3B	142.1	C8—C7—H7B	109.0
H3A—O3—H3B	94	H7A—C7—H7B	107.8
C5—N1—C1	117.2 (5)	C7—C8—C9	111.3 (5)
C5—N1—Mn1	115.0 (3)	C7—C8—H8A	109.4
C1—N1—Mn1	127.7 (4)	C9—C8—H8A	109.4
N1—C1—C2	123.3 (6)	C7—C8—H8B	109.4
N1—C1—H1	118.4	C9—C8—H8B	109.4
C2—C1—H1	118.4	H8A—C8—H8B	108.0
C3—C2—C1	118.4 (6)	C9^iii^—C9—C8	113.9 (5)
C3—C2—H2	120.8	C9^iii^—C9—H9A	108.8
C1—C2—H2	120.8	C8—C9—H9A	108.8
C2—C3—C4	119.7 (6)	C9^iii^—C9—H9B	108.8
C2—C3—H3	120.2	C8—C9—H9B	108.8
C4—C3—H3	120.2	H9A—C9—H9B	107.7
C5—C4—C3	118.3 (6)		
			
O2—Mn1—O1—C6	176.8 (4)	C5—N1—C1—C2	−1.1 (9)
O3—Mn1—O1—C6	89.7 (4)	Mn1—N1—C1—C2	−177.3 (4)
N1—Mn1—O1—C6	2.3 (4)	N1—C1—C2—C3	2.0 (10)
Br1—Mn1—O1—C6	21.6 (8)	C1—C2—C3—C4	−1.6 (10)
Br2—Mn1—O1—C6	−93.2 (4)	C2—C3—C4—C5	0.4 (10)
O3—Mn1—O2—C6^i^	−89.0 (6)	C1—N1—C5—C4	−0.2 (8)
O1—Mn1—O2—C6^i^	−175.9 (6)	Mn1—N1—C5—C4	176.5 (4)
N1—Mn1—O2—C6^i^	−164.0 (5)	C1—N1—C5—C6	179.3 (5)
Br1—Mn1—O2—C6^i^	−2.1 (6)	Mn1—N1—C5—C6	−4.0 (6)
Br2—Mn1—O2—C6^i^	91.3 (5)	C3—C4—C5—N1	0.5 (9)
O2—Mn1—N1—C5	−10.9 (6)	C3—C4—C5—C6	−178.9 (5)
O3—Mn1—N1—C5	−86.0 (4)	Mn1—O1—C6—O2^ii^	174.2 (4)
O1—Mn1—N1—C5	1.3 (4)	Mn1—O1—C6—C5	−5.2 (6)
Br1—Mn1—N1—C5	−174.2 (4)	N1—C5—C6—O2^ii^	−173.3 (5)
Br2—Mn1—N1—C5	92.4 (4)	C4—C5—C6—O2^ii^	6.3 (8)
O2—Mn1—N1—C1	165.3 (4)	N1—C5—C6—O1	6.2 (7)
O3—Mn1—N1—C1	90.3 (5)	C4—C5—C6—O1	−174.3 (5)
O1—Mn1—N1—C1	177.5 (5)	N2—C7—C8—C9	171.0 (4)
Br1—Mn1—N1—C1	2.1 (5)	C7—C8—C9—C9^iii^	176.1 (7)
Br2—Mn1—N1—C1	−91.4 (5)		

Symmetry codes: (i) *x*, −*y*, *z*−1/2; (ii) *x*, −*y*, *z*+1/2; (iii) −*x*, *y*, −*z*+3/2.

### Hydrogen-bond geometry (Å, °)

**Table:**

*D*—H···*A*	*D*—H	H···*A*	*D*···*A*	*D*—H···*A*
N2—H2A···O1^ii^	0.89	2.44	2.949 (6)	117
N2—H2A···O2^ii^	0.89	2.47	3.300 (6)	155
N2—H2B···Br2^ii^	0.89	2.61	3.339 (4)	139
N2—H2C···Br2^iv^	0.89	2.58	3.417 (5)	157
O3—H3A···Br1^v^	0.99	2.28	3.245 (4)	165
O3—H3B···O1^vi^	0.83	2.18	2.961 (5)	157

Symmetry codes: (ii) *x*, −*y*, *z*+1/2; (iv) *x*, *y*, *z*+1; (v) −*x*+1, *y*, −*z*+1/2; (vi) −*x*+1, −*y*, −*z*+1.
